# 2669. Improving Trichomoniasis Screening Among Cis- and Transgender Women with HIV

**DOI:** 10.1093/ofid/ofad500.2280

**Published:** 2023-11-27

**Authors:** Nnamdi Igwe, Yuderka Goris, Berlinda Olivier, Alexandra Abrams-Downey

**Affiliations:** Icahn School of Medicine at Mount Sinai, New York, New York; Icahn School of Medicine at Mount Sinai, New York, New York; Mount Sinai IAM, New York, New York; Icahn School of Medicine at Mount Sinai, New York, New York

## Abstract

**Background:**

In 2020, the *Primary Care Guidance for Persons with HIV* added the recommendation for annual trichomoniasis screening. We identified a low trichomoniasis annual screening rate among cis- and transgender female patients living with HIV at the Institute for Advanced Medicine – Morningside Clinic, a New York State Designated AIDS Center with over 1200 patients with HIV in the Mount Sinai Health System in Manhattan. The primary aim of this prospective quality improvement pilot project was to increase the clinic’s annual trichomoniasis screening rate to 25% over the six-month intervention period.

**Methods:**

A gap analysis identified lack of efficient workflow as the major reason for low trichomoniasis screening. We developed and implemented an intervention as outlined in Figure 1. The intervention began on October 15, 2022. The data was reviewed on April 19, 2023 after the intervention period ended.

**Figure:**
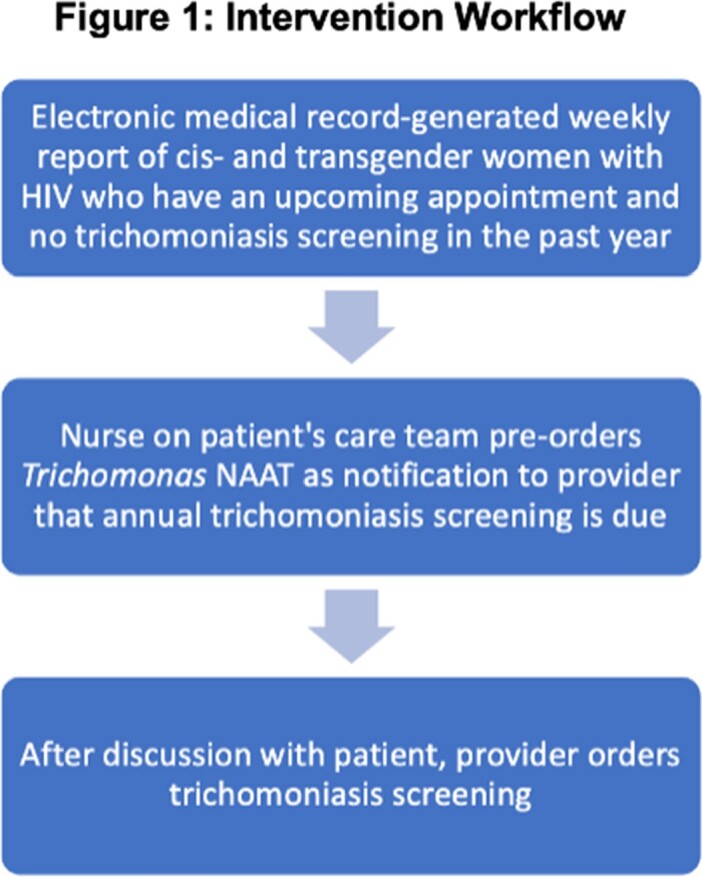

**Results:**

At baseline, there were 423 cis- and transgender women with HIV engaged in care at the Morningside Clinic eligible for annual sexually transmitted infection screening, including trichomoniasis. Pre-intervention screening for trichomoniasis was low at 13.7% (n=58). During the six-month intervention period, there were 280 cis- and transgender women with HIV who required annual trichomoniasis screening and seen at least once for a clinic visit with a primary care provider. Of these 280 eligible patients, 178 were tested for trichomoniasis, a significantly increased screening rate of 63.6%.

**Conclusion:**

The intervention successfully increased the clinic’s annual trichomoniasis screening rate from 13.7% to 63.6% among women with HIV. The observed increase within six months was above our goal and demonstrates the effectiveness of a low-intensity and highly sustainable intervention to improve annual trichomoniasis screening rates among women with HIV. Despite the intervention, there are still a number of patients who remain unscreened for trichomoniasis as well as other sexually transmitted infections such as gonorrhea, chlamydia and syphilis. We will continue this intervention longitudinally and further identify and address barriers to sexually transmitted infection screening among people living with HIV.

**Disclosures:**

**All Authors**: No reported disclosures

